# Pulmonary Kaposi Sarcoma as an Unusual Etiology of Acute Hypoxemic Respiratory Failure in the Era of Highly Active Antiretroviral Therapy: A Case Report

**DOI:** 10.7759/cureus.25014

**Published:** 2022-05-15

**Authors:** Abdelnasir Mohamed, Eltaib Saad, Akram Babkir, Khlwd Khtab, Monzer Abdalla

**Affiliations:** 1 Internal Medicine, AMITA Health Saint Francis Hospital, Evanston, USA; 2 Internal Medicine, University of Khartoum, Khartoum, SDN

**Keywords:** haart, cd4 count, aids-defining diseases, hiv, pulmonary kaposi sarcoma

## Abstract

Kaposi sarcoma (KS) is caused by human herpesvirus 8 (HHV-8). Epidemic KS is described in the human immunodeficiency virus (HIV) population with acquired immune deficiency syndrome (AIDS). It primarily affects the skin, but it may uncommonly disseminate to involve extracutaneous sites such as the gastrointestinal (GI) tract, liver, and lungs.

In this case report, the authors report a 26-year-old homosexual male who was admitted with acute hypoxemic respiratory failure. He was diagnosed with an HIV infection about five months before index presentation, and he was commenced on highly active antiretroviral therapy (HAART). Physical examination was remarkable for diffuse cutaneous nodules over the lower extremities, back, and oropharynx. Chest imaging revealed diffuse bilateral infiltrates, mediastinal adenopathy, and a persistent bilateral pleural effusion. Extensive diagnostic workup was negative for underlying infectious etiology. Transbronchial biopsy demonstrated proliferated spindle cells that stained positive for HHV-8 in keeping with pulmonary KS. Skin biopsies also concurred with the diagnosis of cutaneous KS. Interestingly, the cluster of differentiation 4 (CD4) count was 647 cells/mm^3^, and HIV viral load (VL) was 500 copies/ml. This case demonstrated an atypical natural history of pulmonary KS in an HIV patient as pulmonary and disseminated mucocutaneous KS occurred with a relatively higher CD4 count (≥500 cells/mm^3^). It also reminds pulmonologists and infectious disease specialists to consider pulmonary KS as a differential diagnosis of acute hypoxemic respiratory failure in HIV patients, even in the absence of other clinical and laboratory criteria that define the AIDS stage.

## Introduction

Kaposi sarcoma (KS) is an angioproliferative tumor of the blood and lymphatic vessels, which is strongly associated with human herpesvirus 8 (HHV8), also known as the KS [1]. The cutaneous sites are primarily affected, typically the skin of the lower extremities, face, trunk, and genitalia [2,3]. In addition, visceral organs (GI tract, liver, and lungs) may also be involved in the disseminated form of KS [2,3]. Epidemiologically, there are four distinct variants of KS, classic KS (which is usually prevalent in older men of central European or Mediterranean ancestry), endemic KS (sub-Saharan African patients with neoplastic conditions), iatrogenic KS (in immunosuppressed solid organ transplant hosts), and epidemic KS that is associated with AIDS patients [3-5]. In fact, KS is one of the AIDS-defining diseases, according to the Center for Disease Control and Prevention (CDC) [6].

Herein, the authors described an unusual case of a young homosexual male with HIV infection, while under highly active antiretroviral therapy (HAART) with a recent CD4 count of 647 cells/mm^3^, who was admitted with acute hypoxemic respiratory failure, and he was found to have an extensive pulmonary KS. The occurrence of pulmonary and disseminated cutaneous KS with a relatively higher CD4 count (≥500 cells/mm^3^) in our patient represents a clinical rarity that was seldom reported in the available literature [1,7].

## Case presentation

**Figure 1:**
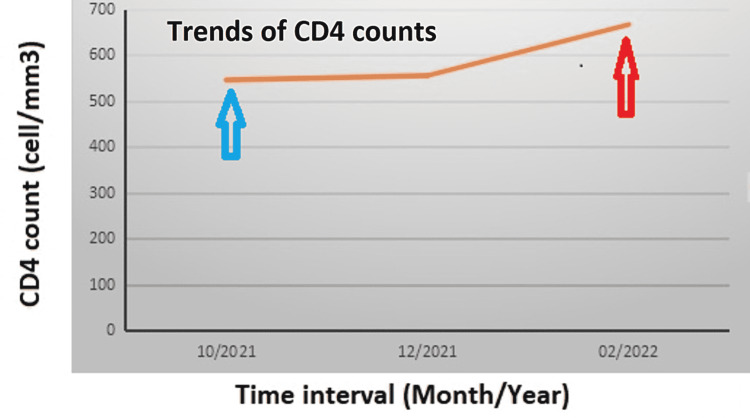
The graph is showing the trends of CD4 count from the initial HIV diagnosis and HAART initiation to the diagnosis of pulmonary and cutaneous KS The blue vertical arrow points to the time of original HIV diagnosis and initiation of ART, and the red vertical arrow indicates the time of diagnosis of pulmonary and mucocutaneous KS. CD4: Cluster of differentiation 4; HAART: highly active antiretroviral therapy; ART: antiretroviral treatment; KS: Kaposi sarcoma.

A 26-year-old African American male patient presented with a non-productive cough and gradually progressive shortness of breath for four weeks that persisted despite a one-week course of amoxicillin followed by a two-week course of doxycycline and injectable ceftriaxone. Symptoms were associated with pleuritic right-sided chest pain. The patient denied fevers or rigors, hemoptysis, night sweating, or unintentional weight loss. He noticed colored skin nodules that first appeared on the lower extremities and then spread to involve his back and upper extremities over weeks preceding the chest complaints. The review of systems was negative for pertinent positive symptoms. He was diagnosed with HIV infection about five months before his current admission at an outside facility, and he was commenced on a fixed-dose HAART (as a combination of bictegravir/emtricitabine/tenofovir alafenamide [Biktarvy], one tablet daily). He disclosed regular participation in unprotected anal intercourse with an HIV-positive male partner who has had multiple male partners. He admitted to abusing cocaine in the past but denied tobacco use. The patient endorsed regular compliance with HAART. Figure 1 summarizes the trends of CD4 count from the initial diagnosis to the index presentation.

General examination revealed a distressed patient. He was afebrile, hemodynamically stable, and initially desaturating to 82% on ambient air, which improved to 94% on 4 L of oxygen through a nasal cannula. There was diffuse lymphadenopathy in anterior cervical, posterior cervical, bilateral submandibular, and axillary groups. Chest examination revealed reduced breathing sounds over both lung bases with diffuse rales over upper and mid zones bilaterally. The lower extremities were edematous with bilateral inguinal lymphadenopathy. Skin examination revealed multiple varied-sized purplish violaceous lesions over both lower extremities, trunk, back, and oropharynx. 

**Figure 2:**
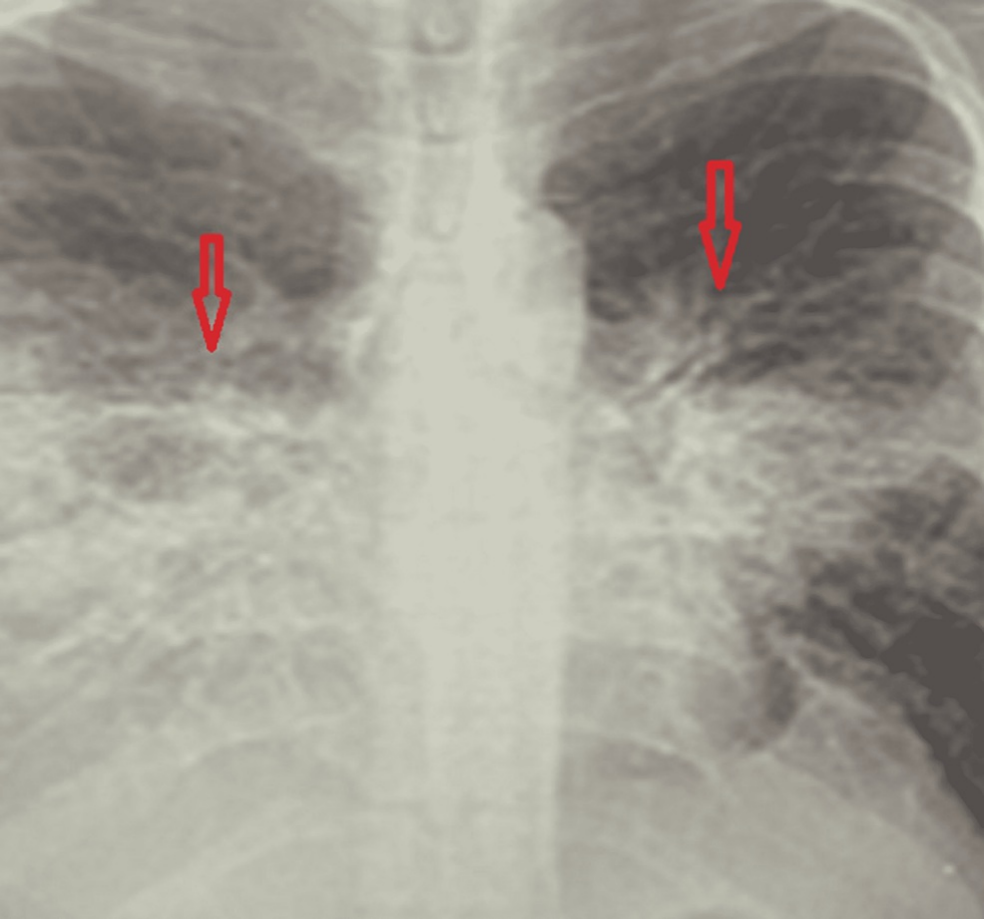
CXR revealing diffuse perihilar interstitial opacities (bilateral vertical arrows) and bilateral pleural effusion worse on the right side CXR: Chest x-ray.

The complete blood count showed normocytic anemia (hemoglobin of 9.5 g/dl) and a normal white cell count. Alkaline phosphatase (ALP) levels were elevated (205 IU/L, normal range of 49-90 IU/L). Chest x-rays (CXRs) revealed diffuse perihilar interstitial opacities and bilateral pleural effusion worse on the right side (Figure 2). No previous CXR was available to compare. The SARS-CoV-2 screening was negative. Blood cultures did not grow any organisms. CD4 count was 647 cell/mm^3^, and the viral load (VL) of HIV was 5,000 copies/ml.

**Figure 3:**
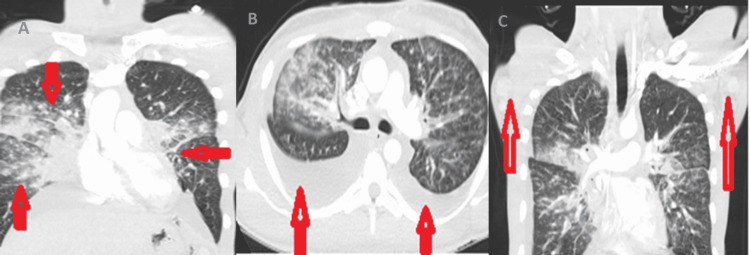
A contrast-enhanced CT chest (A) Coronal image: The arrows show diffuse bilateral airspace opacities concerning atypical multifocal pneumonia. (B) Axial image: The vertical arrows are pointing to bilateral pleural effusions. (C) Coronal image: Vertical arrows point toward the necrotic-looking enlarged axillary lymph nodes that were suspicious of a disseminated infectious or neoplastic process.

Contrast-enhanced CT chest depicted extensive bilateral airspace opacities and large bilateral effusions suspicious of atypical multifocal pneumonia (Figure 3, Panels A and B). There were also mediastinal and necrotic-looking axillary lymph nodes suspicious of a disseminated disease process (Figure 3, Panel C). The differential diagnosis of the clinically correlated radiological appearances included pulmonary tuberculosis with extrapulmonary lymphadenopathy in the setting of the immune reconstitution inflammatory syndrome (IRIS), fungal infections, pneumocystis carinii pneumonia (PCP), and lymphoproliferative disorders. 

QuantiFERON gold was unreactive, and serial morning sputum samples were negative for acid-fast bacilli (AFB). Bilateral thoracocentesis provided symptomatic relief and yielded a serosanguinous exudative fluid with lymphocytic predominate. Gram staining and AFB of the pleural fluid were negative as bacterial cultures and cytological analysis. Urine antigens for fungal infections (histoplasma, coccidioides, and blastomyces) were unrevealing. In addition, aspergillus, cryptococcal, and D-glucan antigens were also negative.

**Figure 4:**
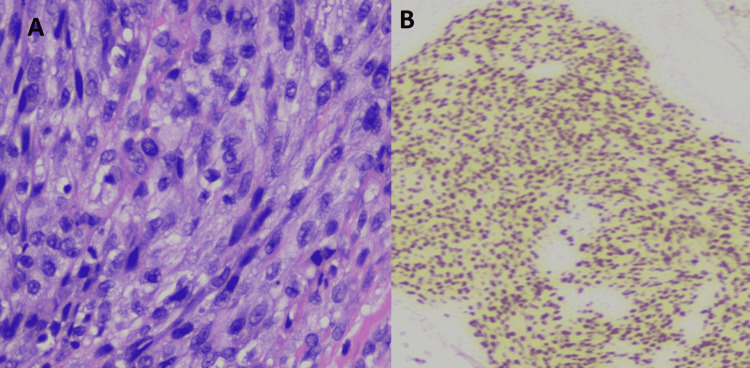
Hematoxylin and eosin (H&E)-stained histopathological image (x40) (A) Transbronchial biopsies showing proliferation of spindle cells with lymphocytic infiltration. (B) Nuclear HHV8 staining of proliferated spindle cells. HHV8: Human herpesvirus 8.

Flexible bronchoscopy showed no tracheal or endobronchial lesions down to the subsegmental level. Bronchoalveolar lavage (BAL) yielded serous aspirate that was negative for PCP (using polymerase chain reaction [PCR]), and bacterial and fungal cultures yielded no growth. Transbronchial biopsies from the lesions of the right upper and middle lobes demonstrated spindle-shaped fibroblast-like proliferated cells (Figure 4) that tested positive for HHV-8 stains in keeping with pulmonary KS. Special stains for PCP, AFB, and fungi were negative. Skin biopsies also concurred with the diagnosis of cutaneous KS.

A diagnosis of pulmonary and mucocutaneous KS in the setting of IRIS was made. Abdominal imaging and endoscopic examination revealed no evidence of visceral KS or other suspicious gastrointestinal AIDS-defining lesions. The patient was continued on his current regime of HAART per the infectious diseases team, and he underwent a series of thoracocentesis under interventional radiology (IR) guidance. Oncology consultation recommended initiating inpatient systemic chemotherapy considering the severity of the pulmonary KS. Doxorubicin liposome 20 mg/m^2^ was administered with good tolerance and was then scheduled every 21 days as an outpatient.

After three weeks, the patient was discharged on ambient air in a stable condition with a pleural catheter in-situ. Follow-up chest imaging at six weeks revealed an interval reduction of bilateral airspace opacities and decreased bilateral pleural effusions, while the mucocutaneous disease remained stable with no new lesions.

## Discussion

Epidemic or AIDS-related KS is primarily prevalent in homosexual and bisexual males in the resource-rich Western countries [1,2,5,7]. However, the gender-related differences are much less pronounced in sub-Saharan Africa and Southeast Asia, where heterosexual females were more relatively affected [4,7]. In the HIV population, the development of KS is strongly related to CD4 counts [8]. In a series of 70 patients with newly diagnosed KS while they were on HAART, the rate ratios for developing KS for patients with CD4 counts < 200, 200-349, and 350-499 cells/mm^3^ were 18.9, 3.6, and 4.1, respectively, compared to those with ≥500 cells/mm^3^ [8]. Therefore, the occurrence of pulmonary and mucocutaneous KS with a relatively higher CD4 count in our case represents a clinical rarity [8]. As combination-based HAART is becoming more readily available, the incidence of KS is significantly declining, and survival is improving [8]. Interestingly, the incidence of KS is often higher during the first few months of initiating HAART, and this observation may be attributable to the unmasking of KS by IRIS [7,8].

The mucocutaneous lesions of KS appear most frequently on the lower extremities, trunk, genitalia, and oral mucosa [9]. Furthermore, lower extremity edemas observed in our patient may be explained by lymphadenopathy-related vascular obstruction and the local effects of inflammatory cytokines [9]. Bacillary angiomatosis is a great clinicopathological mimicker of cutaneous KS; hence, the histopathological examination of tissue biopsy is crucial to differentiate between the two entities [8,9]. The three hallmark histological features of KS are chronic inflammation, fibroblast-like spindle cell proliferation, and angiogenesis with aberrant neovascularization of small blood vessels [9].

Pulmonary KS is usually associated with extensive mucocutaneous disease in more than 90% of cases [10]. Nevertheless, there were reports of pulmonary disease in the absence of mucocutaneous lesions [9,10]. Symptoms of the parenchymal disease include dry cough, dyspnea, and low-grade fevers that progress over a few weeks and eventually manifest as an acute hypoxemic respiratory failure as demonstrated in our patient [1,10]. In addition, hemoptysis may denote endobronchial involvement [10].

Chest radiograph commonly shows septal infiltration and patchy reticular opacities (60%), followed by ill-defined nodular densities (25%) [11,12]. While CT chest usually reveals hilar densities that extend into peribronchial and perivascular spaces in a characteristic septal or nodular pattern with concomitant pleural effusions [12]. CT imaging also helps evaluate other concurrent infectious or neoplastic processes [1]. It is important to note that these clinical and radiological features can almost be indistinguishable from other opportunistic infections in AIDS (such as atypical bacteria, mycobacteria, and fungi) as well as AIDS-related neoplastic conditions (like pulmonary non-Hodgkin’s lymphoma, primary effusion lymphoma, and HHV-8-associated Castleman disease) [11].

The presumptive diagnosis of pulmonary KS is clinically based on the epidemiology (high-risk groups, i.e., men who have sex with men, MSM), the severity of immunodeficiency, presence of mucocutaneous lesions, probable radiological appearances, and exclusion of other infectious or neoplastic etiologies (as shown in our patient). The diagnosis is usually confirmed when characteristic endobronchial lesions (such as violaceous or bright red maculopapular lesions) are identified during bronchoscopy along the mucosa of the lower bronchi and less likely trachea [8,9]. Occasionally, transbronchial and endobronchial biopsies are positive for the HHV-8 virus, but their diagnostic yield is generally low [13]. PCR-based detection of HHV-8 in BAL aspirate can also be diagnostic for pulmonary KS with high sensitivity (80%) [11]. Notably, establishing the diagnosis of pleural KS in the absence of parenchymal disease is difficult. Pleural cytology and pleural biopsies do not help diagnose pleural KS, but they may be useful in excluding other etiologies [12,13].

The principal management of pulmonary KS is HAART, which has led to a significant reduction in the incidence of the disease and altered its natural history with improved survival [7,12]. Concomitant systemic chemotherapy with cytotoxic agents is indicated when there is evidence of rapid disease progression or advanced visceral disease [7,8]. Discontinuation of systemic corticosteroids was associated with regression of KS lesions in patients on steroids therapy for concomitant HIV-related conditions (such as PCP pneumonia) [10]. Management of KS-related large pleural effusion can be challenging, as shown in our case, and it entails a combination of HAART and cytotoxic chemotherapy with repeated thoracocentesis [14]. In addition, placement of an indwelling pleural catheter may be required for recurrent effusion [14].

## Conclusions

Pulmonary KS should be considered a possible differential diagnosis of acute hypoxemic respiratory failure in HIV patients, even in the absence of other clinical and laboratory criteria that define the AIDS stage. The occurrence of pulmonary and disseminated mucocutaneous KS with a relatively high CD4 count is a clinical rarity that was seldom reported. Multidisciplinary management with the early involvement of infectious disease specialists and pulmonologists is essential to achieve a better outcome in HIV patients with acute hypoxemic respiratory failure secondary to pulmonary KS.
